# Continuous Renal Replacement Therapy Versus Intermittent Hemodialysis for Renal Prognosis in Elderly Patients With Acute Kidney Injury

**DOI:** 10.1155/ijne/8899604

**Published:** 2025-03-11

**Authors:** Enhui Li, Linlin Zhang, Yikai He, Huipeng Ge, Rong Tang, Jinbiao Chen, Yong Zhong, Xiangning Yuan, Weiwei Zhang, Yizi Gong, Xiangcheng Xiao

**Affiliations:** ^1^Department of Nephrology, Xiangya Hospital, Central South University, Changsha, Hunan, China; ^2^Department of Nephrology, Nanyang Central Hospital, Nanyang, Henan, China; ^3^Department of Nephrology and Rheumatology, First Hospital of Changsha, Changsha, Hunan, China

**Keywords:** acute kidney injury, elderly patients, modalities, prognosis, renal replacement therapy

## Abstract

**Background:** Continuous renal replacement therapy (CRRT) and intermittent hemodialysis (IHD) represent two common modes of renal replacement therapy (RRT) for elderly patients with acute kidney injury (AKI), but their clinical effectiveness is debated. This study aimed to compare the impact of CRRT and IHD on renal prognosis in elderly patients with AKI by analyzing their clinical data.

**Methods:** The retrospective study population included elderly patients admitted to Xiangya Hospital between 2018 and 2022, who required RRT for AKI. Patients were separated into two cohorts based on the original RRT modes (CRRT or IHD). In our study, the primary outcome was recovery of renal function at discharge and the secondary outcome was RRT dependency rate at 90 days. A multivariate logistic regression model was constructed for the purpose of comparing the impact of CRRT and IHD on renal prognosis.

**Results:** The mortality rate at the time of patient discharge was significantly elevated in the CRRT cohort relative to the IHD cohort (49.6% vs. 2.1%, *p* < 0.001). However, for the 155 patients who survived at discharge, the analysis revealed no statistically significant discrepancy in renal recovery across the two groups (40.3% vs. 59.7%, *p* = 0.694). Multivariate logistic regression analysis showed no statistically meaningful distinction among the CRRT and IHD groups concerning renal function recovery at discharge. Nevertheless, in comparison with IHD, CRRT reduced the risk of RRT dependence at 90 days.

**Conclusions:** Our study indicated that CRRT and IHD have comparable effects on renal recovery at discharge in elderly patients with AKI who require RRT. However, in comparison with IHD, CRRT was linked to a diminished likelihood of requiring RRT at 90 days.

## 1. Introduction

Acute kidney injury (AKI) is characterized by significant mortality and adverse prognostic outcomes [1]. Recent epidemiological data indicate a gradual rise in AKI prevalence, with up to 20% of inpatients being affected, primarily among the elderly [2, 3]. Advanced age predisposes individuals to AKI due to underlying complications and age-related changes in renal physiology [4]. The metabolic and inflammatory cascades triggered by AKI exacerbate renal injury, significantly impacting the patient's prognosis [5]. Acute metabolic disorders, inflammation, and oxidative stress caused by AKI can further exacerbate kidney damage, significantly affecting the prognosis and outcomes of elderly patients. Schmitt et al. observed a staggering 31.1% incidence of nonrecovery or deterioration to end-stage renal disease in AKI patients aged over 65 [6].

Renal replacement therapy (RRT) is of great importance in removing toxins, maintaining fluid balance, and correcting acid–base and electrolyte imbalances [7, 8]. It can significantly improve clinical outcomes and reduce mortality in AKI patients, making it a primary treatment modality for AKI [9, 10]. Therefore, timely and effective RRT is essential for elderly AKI patients.

Continuous renal replacement therapy (CRRT) and intermittent hemodialysis (IHD) are primary RRT modalities for AKI management [11]. CRRT is the recommended treatment for patients with AKI who are critically ill, given its capacity to continuously and gradually remove solutes [12]. IHD, which can rapidly clear fluids and toxins and is cost-effective, is also commonly used for AKI treatment, particularly in hemodynamically stable patients [13, 14].

Previous studies comparing the efficacy of CRRT and IHD on the recovery of renal function in AKI patients have been inconclusive. Some research suggests that CRRT may be more beneficial for renal recovery in AKI patients. For instance, a multicenter retrospective cohort study by Bonnassieux et al. included nearly 60,000 ICU patients requiring RRT. They found that the selection of IHD was identified as a factor contributing to a reduced probability of renal recovery at discharge [15]. Similarly, Wald et al. assessed the effects of CRRT versus IHD on renal outcomes in critically ill AKI survivors, reporting higher 90-day dialysis dependence in the IHD cohort compared to the CRRT cohort [16]. Furthermore, the study included a three-year median follow-up period, during which the CRRT cohort exhibited a 25% decreased risk of long-term dialysis dependence in comparison to the IHD cohort.

However, other studies have shown no meaningful distinction across CRRT and IHD regarding renal outcomes in AKI patients. A randomized controlled trial (RCT) involving 360 AKI patients from 21 ICU centers found no discrepancy in renal recovery rates during hospitalization among the CRRT and IHD groups [17]. Liang et al. conducted a retrospective cohort study of 1338 stage 3 AKI patients requiring RRT, and their multivariate analysis indicated no statistical difference between CRRT and IHD concerning 90-day renal survival and 365-day dialysis dependence rates [18].

It is therefore unclear whether one RRT modality is more effective than the other with regard to renal outcomes in the treatment of AKI. Furthermore, most previous clinical studies comparing the efficacy of different RRT modalities have focused on adult populations, with a lack of dedicated research on elderly AKI patients. Consequently, the objective of the study is to retrospectively analyze the clinical data of elderly AKI patients requiring RRT, comparing their clinical characteristics and renal outcomes based on the RRT modality used, whether CRRT or IHD. The goal is to provide insights into the optimal RRT modality selection for elderly AKI patients.

## 2. Methods

### 2.1. Study Patients

This study included elderly inpatients diagnosed with AKI requiring RRT treatment at Xiangya Hospital of Central South University from February 2018 to February 2022. Inclusion criteria included (1) age ≥ 60 years, regardless of gender or race; (2) AKI diagnosis follows the 2012 Kidney Disease: Improving Global Outcomes (KDIGO) guidelines utilizing serum creatinine levels to classify AKI stages [12], encompassing stages 1, 2, and 3; and (3) receiving either IHD or CRRT. The exclusion criteria included (1) pre-existing AKI (CKD) necessitating maintenance hemodialysis; (2) kidney transplant recipients; (3) survival less than 48 h post-AKI onset; (4) adjunctive blood purification therapies; (5) obstructive or prerenal AKI cases with spontaneous recovery; (6) tumor patient; and (7) incomplete data. This study was approved by the Clinical Medical Ethics Committee of Xiangya Hospital (approval number K202204236).

### 2.2. Data Collection

Once the study subjects had been selected by the pre-established inclusion and exclusion criteria, the following data were collected: (1) demographic information, including patient age and gender; (2) clinical data, including baseline serum creatinine and departmental source; and (3) treatment-related data, including RRT modality and mode switching.

### 2.3. Study Grouping and Outcomes

Patients were separated into two cohorts, the CRRT cohort and the IHD cohort, following their initial choice of RRT modality. The primary outcome assessed was renal function recovery at discharge. The secondary outcome was the necessity for maintenance dialysis.

### 2.4. Study Definitions

Definitions used in this study are as follows. (1) Baseline creatinine: The lowest creatinine level within 7 days before elevation, or if unavailable, within 365 days [19]. If no data, eGFR was assumed to be 75 mL/min/1.73 m^2^ [12], and baseline creatinine was estimated using the CKD-EPI formula. (2) AKI staging (2012 KDIGO guidelines) [12]: Stage 1: 1.5–1.9-fold increase from baseline or > 26.5 μmol/L (0.3 mg/dL) increase; Stage 2: 2.0-2.9-fold increase from baseline; and Stage 3: ≥ 3-fold increase from baseline or ≥ 353.6 μmol/L (4.0 mg/dL). (3) Oliguria and anuria: Oliguria: Urine output < 400 mL/24 h without diuretics; Anuria: Urine output < 100 mL/24 h without diuretics. (4) RRT modality: CRRT was performed bedside with a blood flow rate of 150–180 mL/min for 8–24 h per day, including continuous venovenous hemofiltration (CVVH), continuous venovenous hemodialysis (CVVHD), and continuous venovenous hemodiafiltration (CVVHDF); IHD was conducted in the hemodialysis unit with a blood flow rate of 180–220 mL/min, with each session lasting approximately 4 h (5) RRT duration: The duration of a single RRT treatment. (6) Renal function recovery at discharge [15]: Patients are considered to have recovered renal function when they are alive at discharge and have been weaned off CRRT. Conversely, if they are alive at discharge but still dependent on CRRT, their renal function is regarded as not recovered. (7) Maintenance dialysis: Required if alive on the 90th day post-CRRT initiation and still dependent on dialysis [16].

### 2.5. Statistical Analyses

Continuous variables with normal distribution were presented as mean ± standard deviation; non-normal variables were presented as median (interquartile range). Categorical variables were expressed as frequencies (percentages). The *t*-test compared normally distributed continuous variables, and the Mann–Whitney *U* test compared non-normal ones. Categorical variables were compared using Pearson's chi-square or Fisher's exact test. Significant variables in univariate analyses were included in a multivariate logistic regression to assess the impact of CRRT versus IHD on renal prognosis. Statistical analysis was performed using SPSS 26.0, with *p* < 0.05 considered significant.

## 3. Results

### 3.1. Baseline Characteristics and Treatment of Patients in the CRRT and IHD Cohorts

**Figure 1:**
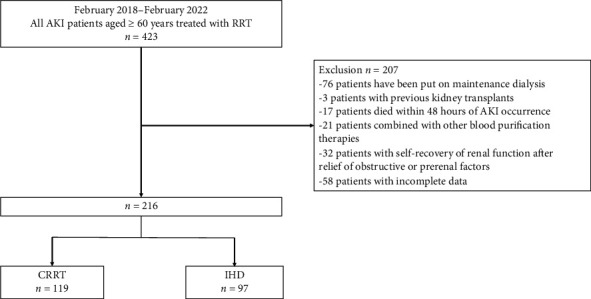
Graphical representation of inclusion exclusions in this study. AKI: acute kidney injury; RRT: renal replacement therapy; CRRT: continuous renal replacement therapy; IHD: intermittent hemodialysis.

A total of 216 patients were enrolled in the study under the established inclusion and exclusion criteria (Figure 1), with 119 in the CRRT cohort and 97 in the IHD cohort. The baseline characteristics and treatment details of the patients are shown in Tables 1 and 2. The mean age of the patients was 67 years, with 149 (69.0%) being male. The median duration of RRT was 47 h, and the overall hospital mortality rate was 28.2%. A comparison of the IHD group with the CRRT group revealed that the former were older and had a higher prevalence of diabetes, cardiovascular disease, and CKD. Most patients in the CRRT group were from the ICU (63.9%), whereas most patients in the IHD group were from general medical wards (71.1%). Patients in the CRRT group had higher APACHE II scores at the initiation of RRT, a higher incidence of cardiovascular events, and an increased requirement for vasopressor support and mechanical ventilation compared to the IHD group. Additionally, the CRRT group had more severe anemia, greater cardiac and liver dysfunction, higher risk of coagulopathy, and elevated infection and inflammation markers, in comparison to the IHD group.

The CRRT group had longer blood purification treatment durations, higher treatment costs, and significantly longer hospital stays compared to the IHD group (456 h vs. 360 h). However, no notable discrepancies were observed regarding dialysis-related adverse events between the two groups, such as filter clotting and allergic reactions. 21 patients underwent a modality transition during treatment, 19 in the CRRT group and 5 in the IHD group. Patients changed their dialysis treatment pattern due to a change in their condition (improvement or deterioration), but in most cases, the change occurred only once. The mean time to pattern change for patients in the CRRT group was 18 days after the first CRRT, and for patients in the IHD group, the mean time to pattern change was 12 days after the first IHD. The mortality rate among patients who underwent CRRT was significantly greater than that observed among patients who underwent IHD.

### 3.2. Baseline Characteristics and Treatment of Patients With and Without Renal Function Recovery at Discharge

Of the 155 patients surviving to discharge, 77 (49.7%) demonstrated recovery of renal function, while 78 (50.3%) exhibited no such recovery. The baseline characteristics and treatment details of these patients are shown in Tables 3 and 4. About the baseline characteristics, patients exhibiting a lack of renal function recovery were older, had a higher prevalence of pre-existing CKD, higher baseline serum creatinine levels, and more severe anemia compared to those who demonstrated renal function recovery. Patients with unresolved renal dysfunction required a significantly longer duration of RRT (43 h vs. 37 h, *p* = 0.043).

### 3.3. Comparison of the Effects of CRRT and IHD on the Recovery of Renal Function at Hospital Discharge

Univariate logistic regression analysis revealed that upon comparison with IHD, CRRT was found to exert no statistically discernible influence on the recovery of renal function at the time of discharge (OR = 0.878, 95% CI 0.460–1.677, *P* = 0.694). The covariates affecting renal function recovery at discharge included age, baseline creatinine, pre-existing CKD, and hemoglobin levels (Table 5). Multivariate logistic regression models were constructed incorporating the significant covariates (Table 6). The results of both models indicated that no statistically discernible discrepancy existed between the impact of CRRT and IHD on renal function recovery at discharge. Additionally, Model 1 showed that older age correlated with an elevated risk of renal function nonrecovery at the time of discharge, and a history of CKD increased this risk. Model 2 found that for every 10 g/L increase in hemoglobin before the initial RRT, the risk of nonrecovery of renal function at discharge decreased by 25%.

### 3.4. Comparison of the Effects of CRRT and IHD on Maintenance Dialysis

**Figure 2:**
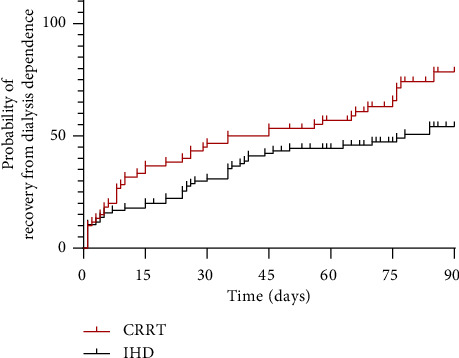
Inverted Kaplan–Meier curves for dialysis dependence among survivors of AKI. The red line represents patients treated with CRRT and the black line represents patients treated with IHD. Log-rank (Mantel–Cox) test: *p* = 0.0136; Gehan–Breslow–Wilcoxon test: *p* = 0.0432. AKI: acute kidney injury; CRRT: continuous renal replacement therapy; IHD: intermittent hemodialysis.

Of the 155 patients who survived to discharge, follow-up was conducted until the 90th day after RRT. Among them, 28 patients did not complete the follow-up period, 55 patients died, and 72 patients were still alive. Among the surviving patients, 27 (37.5%) required maintenance dialysis, with 23 (85.2%) from the IHD group and 4 (14.8%) from the CRRT group. Univariate analysis revealed a statistically noteworthy discrepancy between CRRT and IHD concerning the need for maintenance dialysis (*p*=0.045). Other covariates affecting the need for maintenance dialysis included age and white blood cell count (Table 7). Multivariate logistic regression models were constructed incorporating these significant covariates (Table 8). The Kaplan–Meier curves for recovery from dialysis dependence among survivors at discharge are shown in Figure 2. The findings demonstrated that, in comparison to IHD, CRRT was associated with a markedly reduced likelihood of requiring maintenance dialysis in elderly patients with AKI. Furthermore, an increased risk of requiring maintenance dialysis was observed in individuals of advanced age.

### 3.5. Sensitivity Analysis

In light of the potential for disease progression to result in the necessity for dialysis, even among patients presenting with pre-existing CKD without an acute exacerbation, sensitivity analysis was conducted by excluding such patients to validate the accuracy of the study results. The univariate analysis indicated that there was no statistically considerable distinction in the effectiveness of CRRT in contrast to IHD on renal function recovery at discharge. Age and hemoglobin levels were identified as distinct covariates (Table 9). Subsequently, a multivariate logistic regression model was constructed, incorporating age, RRT modality, AKI stage, and hemoglobin levels (Table 10), which still showed no statistically significant difference in the comparative influence of CRRT and IHD on the recovery of renal function at discharge (*p* = 0.143). The risk of nonrecovery of renal function at discharge was found to be higher in patients with increasing age. A 20.4% reduction in the risk of nonrecovery of renal function at discharge was observed with each 10 g/L increase in hemoglobin.

## 4. Discussion

This study retrospectively analyzed the clinical data of 216 elderly AKI patients requiring RRT over four years, comparing CRRT and IHD impacts on renal outcomes. Patients treated with CRRT were older and had more comorbidities, severe illness, higher vasopressor and ventilator dependence, and higher in-hospital mortality rates. IHD patients had milder conditions and lower mortality rates. A comparison of the outcomes in kidney function recovery at discharge revealed no statistically substantial distinction between the two treatment modalities, namely, CRRT and IHD. However, the incidence of requiring maintenance dialysis was found to be diminished in patients undergoing CRRT in comparison to those undergoing IHD.

CRRT provides slow, continuous, and gentle renal support for critically ill patients, which is closer to physiological conditions. As early as 2012, the KDIGO guidelines on AKI recommended that CRRT be preferred over IHD for AKI patients with hemodynamic instability [12]. However, CRRT has its drawbacks, such as its high cost and increased risk of bleeding [13]. IHD, however, utilizes higher flow rates to maintain fluid, electrolyte, and acid–base balance, which makes it more suitable for hemodynamically stable AKI patients requiring rapid toxin clearance or correction of severe hyperkalemia [20]. Gaudry et al. found higher mortality in CRRT patients with low Sequential Organ Failure Assessment (SOFA) scores compared to IHD, suggesting milder cases should opt for IHD [21]. Our study also identified that patients in the CRRT cohort exhibited more severe illness, elevated APACHE II scores, and increased rates of mechanical ventilation and vasopressor utilization, which collectively resulted in a heightened mortality rate compared to the IHD cohort. This may be attributed to the observation that individuals with more serious conditions may experience hemodynamic instability, resulting in an initial preference for CRRT as a form of treatment.

In our study, the in-hospital mortality rate for the CRRT group was 49.6%, whereas for the IHD group, it was only 2.1%, showing a significant statistical difference. Similarly, Guérin et al. also demonstrated a marked disparity in mortality rates between the groups (79.4% vs. 58.8%) [22]. However, this does not imply a direct association between CRRT and higher in-hospital mortality rates, since this disparity in mortality rates is also influenced by various confounding variables, including age and the gravity of the patient's condition. The available evidence does not support the hypothesis that there is a statistically meaningful disparity in the efficacy of the two treatment modalities on the survival of patients with AKI [23]. The results of the RCTs conducted by Vinsonneau et al. demonstrated that statistically considerable differences in the 28-day, 60-day, and 90-day survival rates between CRRT and IHD were absent among patients with AKI [17].

In previous research, the concept of renal function recovery during hospitalization in AKI patients has been proposed and demonstrated to be associated with long-term prognosis [24]. In our study, 49.7% of surviving AKI patients had recovered renal function by the time of discharge, consistent with Fiorentino and Itenov's findings [25].

This study assessed the impact of two RRT modalities on renal outcomes in elderly patients with AKI. Our results indicate that different RRT modalities (CRRT vs. IHD) are not statistically correlated with renal function recovery at discharge in elderly AKI patients, consistent with sensitivity analysis results excluding elderly AKI patients with pre-existing CKD. This is consistent with previous RCT results from Vinsonneau et al., who focused on adult AKI patients [17].

Additionally, compared to IHD, CRRT may reduce the risk of elderly AKI patients requiring maintenance dialysis. This is similar to findings from retrospective observational studies conducted by Wald et al. and Bell et al. [16, 26]. The mechanism by which CRRT reduces maintenance dialysis risk is not yet clear. CRRT's gradual and continuous control of volume may reduce the risk of hypovolemia and hypotension, thereby potentially decreasing the likelihood of ischemic renal injury caused by RRT treatment [26]. Conversely, IHD is linked to hemodynamic instability during dialysis. Studies have demonstrated a rise in oxygen consumption and a decrease in cardiac output within the initial hour of initiating IHD [27]. Additionally, it may be associated with the function of CRRT in clearing inflammatory factors and reducing the risk of unresolved renal inflammation [28, 29].

Our findings indicated that an elevated age correlated with an augmented risk of unfavorable renal outcomes. This may be related to age-related physiological changes in the kidneys. In the elderly, both renal structure and function undergo age-related changes, with kidney weight decreasing by 20%–30% and renal blood flow decreasing by 10% every decade after the age of 70 [30]. Additionally, older adults have increased body fat, reduced plasma volume, multiple comorbidities, and often require multiple medications. These changes make elderly kidneys more sensitive to various injurious stimuli and reduce their repair capacity [31]. As our study demonstrated, pre-existing CKD also increases the risk of nonrecovery of renal function at discharge in elderly AKI patients. We found that for every 10 g/L increase in hemoglobin before the first RRT session, the risk of nonrecovery of renal function at discharge decreased by 25%, suggesting that correcting anemia may improve renal outcomes in elderly AKI patients. Evidence indicates that anemia is associated with vascular damage, congestive heart failure, increased need for RRT, and higher mortality in elderly patients [32]. This may be due to anemia-induced organ ischemia, chronic inflammation, and increased cardiac output [33, 34].

In this investigation, we focused our analysis on elderly patients with AKI necessitating RRT. To evaluate the primary outcomes, we devised multivariable regression models that incorporated a variety of covariates and conducted sensitivity analysis to validate the robustness of our conclusions. Nonetheless, it is imperative to recognize several inherent limitations. (1) The study is retrospective, conducted at a single center, and comprises a modest cohort size. (2) Patients initially allocated to the CRRT group presented with more severe baseline conditions compared to those in the IHD group, which could have influenced the analysis and interpretation of results. Hence, it is imperative to conduct future multicenter and large-scale prospective studies to more comprehensively explore the comparative effects of various RRT modalities on renal outcomes in elderly AKI patients and to delineate the prognostic significance of RRT initiation timing within this particular demographic.

## 5. Conclusions

In summary, elderly AKI patients initially treated with CRRT were older, had more comorbidities, and were more severely ill compared to those treated with IHD, resulting in a higher in-hospital mortality rate. For elderly AKI patients receiving RRT, there was no significant difference between CRRT and IHD in terms of renal function recovery at discharge. However, CRRT was associated with a lower risk of requiring maintenance dialysis compared to IHD. Additionally, increasing age, pre-existing CKD, and anemia correlated with a higher risk of poor renal outcomes in these patients.

## Figures and Tables

**Table 1:** Baseline characteristics of patients in the CRRT and IHD groups.

Variant	Total (*n* = 216)	CRRT group (*n* = 119)	IHD group (*n* = 97)	*p* value
Male (*n*, %)	149 (69.0)	85 (71.4)	64 (65.9)	0.389
Age (years)	67 (63–72)	70 (65–77)	65 (62–71)	< 0.001⁣^∗∗^
Section source (*n*, %)				
Internal medicine	98 (45.4)	29 (24.4)	69 (71.1)	< 0.001⁣^∗∗^
Surgery	13 (6.0)	9 (7.6)	4 (4.1)	
Emergency	29 (13.4)	5 (4.2)	24 (24.7)	
ICU	76 (35.2)	76 (63.9)	0 (0.0)	
Underlying disease (*n*, %)				
Hypertension	133 (61.6)	74 (62.2)	59 (60.8)	0.838
Diabetes mellitus	50 (23.1)	38 (31.9)	12 (12.4)	0.001⁣^∗∗^
Cardiovascular disease	101 (46.8)	67 (56.3)	34 (35.1)	0.002⁣^∗∗^
CKD	80 (37.0)	57 (47.9)	23 (23.7)	< 0.001⁣^∗∗^
Smoking history (*n*, %)	94 (43.5)	54 (45.4)	40 (41.2)	0.541
Drinking history (*n*, %)	46 (21.3)	23 (19.3)	23 (23.7)	0.434
Baseline blood creatinine (μmol/L)	99 (86–160)	110 (90–167)	92 (75–142)	0.004⁣^∗∗^
AKI pathogenic factors (*n*, %)				
Insufficient renal perfusion	41 (19.0)	35 (29.4)	6 (6.2)	< 0.001⁣^∗∗^
Primary or secondary glomerular disease	54 (25.0)	13 (10.9)	41 (42.3)	
Post-surgical kidney injury	18 (8.3)	16 (13.4)	2 (2.1)	
Drug-induced renal injury	35 (16.2)	10 (8.4)	25 (25.8)	
Severe infection or sepsis	46 (21.3)	33 (27.7)	13 (13.4)	
Other	22 (10.2)	12 (10.1)	10 (10.3)	
AKI stages (*n*, %)				
Stage 1	44 (20.4)	42 (35.3)	2 (2.1)	< 0.001⁣^∗∗^
Stage 2	43 (19.9)	30 (25.2)	13 (13.4)	
Stage 3	129 (59.7)	47 (39.5)	82 (84.5)	
APACHE II score (points)	18 (11–28)	24 (21–31)	11 (9–21)	< 0.001⁣^∗∗^
Cardiovascular accident (*n*, %)	51 (23.6)	46 (38.7)	5 (5.2)	< 0.001⁣^∗∗^
Vasoactive drug (*n*, %)	61 (28.2)	57 (47.9)	4 (4.1)	< 0.001⁣^∗∗^
Mechanical ventilation (*n*, %)	70 (32.4)	66 (55.5)	4 (4.1)	< 0.001⁣^∗∗^
Urination (*n*, %)				
Anuria	67 (31.0)	37 (31.1)	30 (30.9)	0.078
Oliguria	101 (46.8)	62 (52.1)	39 (40.2)	
Normal	48 (22.2)	20 (16.8)	28 (28.9)	
Leukocyte (× 10^9^/L)	9.8 (7.2–14.2)	12.0 (8.4–16.0)	8.5 (6.7–11.6)	< 0.001⁣^∗∗^
Hemoglobin (g/L)	91.5 (75.3–113.0)	84.0 (74.0–102.0)	103.0 (83.0–125.0)	< 0.001⁣^∗∗^
Platelet (× 10^9^/L)	154 (94–229)	141 (74–205)	171 (108–239)	0.044⁣^∗^
Neutrophil (× 10^9^/L)	8.2 (5.5–12.0)	9.5 (6.6–14.0)	6.1 (4.7–9.8)	< 0.001⁣^∗∗^
Lymphocyte (× 10^9^/L)	0.8 (0.5–1.2)	0.7 (0.5–1.1)	1.1 (0.7–1.35)	< 0.001⁣^∗∗^
Hematuria (*n*, %)	111 (51.4)	62 (52.1)	49 (50.5)	0.452
Proteinuria (*n*, %)	131 (60.6)	62 (52.1)	69 (71.1)	0.024⁣^∗^
Albumin (g/L)	29.72 ± 6.25	29.42 ± 4.73	30.09 ± 7.72	0.455
Glutamic-pyruvic transaminase (U/L)	21.7 (10.5–57.3)	31.4 (12.1–134.0)	16.2 (10.2–34.7)	0.004⁣^∗∗^
Glutamic oxalacetic transaminase (U/L)	38.8 (21.3–89.6)	57.8 (26.9–224.0)	25.0 (17.5–44.3)	< 0.001⁣^∗∗^
Urea nitrogen (mmol/L)	25.7 (16.9–37.3)	27.4 (16.3–40.1)	24.5 (16.9–35.1)	0.321
Serum creatinine (μmol/L)	556 ± 320	425 ± 267	716 ± 307	< 0.001⁣^∗∗^
Uric acid (μmol/L)	544.0 (396.7–709.1)	539.2 (407.8–747.2)	548.9 (392.1–662.1)	0.543
Blood potassium (mmol/L)	4.42 (3.91–5.04)	4.57 (4.06–5.19)	4.19 (3.82–4.86)	0.005⁣^∗∗^
Blood sodium (mmol/L)	140 (136–145)	142 (136–148)	139 (135–142)	0.001⁣^∗∗^
Blood chloride (mmol/L)	102.6 (96.9–106.1)	103.4 (96.8–106.4)	102.1 (97.0–105.9)	0.432
Blood calcium (mmol/L)	2.02 (1.87–2.16)	2.04 (1.84–2.17)	1.98 (1.84–2.12)	0.082
Carbon dioxide (mmol/L)	18.9 (15.1–22.8)	19.9 (15.0–24.3)	18.0 (15.2–20.9)	0.16
Anion gap (mmol/L)	18.1 (14.8–22.4)	17.9 (13.1–23.1)	18.3 (15.8–21.6)	0.255
Blood phosphorus (mmol/L)	1.71 (1.29–2.27)	1.56 (1.14–2.22)	1.86 (1.48–2.34)	0.007⁣^∗∗^
Blood magnesium (mmol/L)	0.93 (0.82–1.09)	0.93 (0.82–1.13)	0.94 (0.83–1.06)	0.481
Blood glucose (mmol/L)	6.55 (4.94–8.96)	7.73 (5.85–10.8)	5.34 (4.58–6.80)	< 0.001⁣^∗∗^
Triglyceride (mmol/L)	1.50 (1.08–2.08)	1.40 (1.03–1.95)	1.69 (1.15–2.20)	0.026⁣^∗^
Cholesterol(mmol/L)	3.78 (2.92–4.79)	3.34 (2.55–4.36)	4.20 (3.10–5.32)	0.001⁣^∗∗^
High density lipoprotein (mmol/L)	0.91 (0.70–1.18)	0.84 (0.65–1.06)	0.99 (0.80–1.29)	0.002⁣^∗∗^
Low density lipoprotein (mmol/L)	2.35 (1.79–3.16)	2.14 (1.68–2.81)	2.64 (1.92–3.35)	0.007⁣^∗∗^
Lactate dehydrogenase (U/L)	345.9 (250.4–542.5)	418.0 (280.0–816.0)	292.8 (207.9–345.9)	< 0.001⁣^∗∗^
Creatine kinase (U/L)	103.0 (54.9–286.4)	159.8 (56.4–634.8)	72.4 (52.5–110.6)	0.002⁣^∗∗^
Creatine kinase isoenzyme (U/L)	17.1 (11.8–29.2)	19.3 (11.8–48.3)	15.4 (11.8–20.4)	0.010⁣^∗^
Myoglobin (μg/L)	219.1 (95.9–483.8)	336.1 (135.8–1033.4)	103.6 (77.3–219.1)	< 0.001⁣^∗∗^
Prothrombin time (s)	13.8 (12.7–16.2)	14.9 (13.3–19.2)	13.2 (12.3–14.2)	< 0.001⁣^∗∗^
Activated partial thromboplastin time (s)	37.5 (30.8–45.4)	38.0 (30.0–49.0)	35.9 (32.7–42.2)	0.808
D-dimer (mg/L)	1.34 (0.77–2.75)	1.90 (0.83–4.16)	1.04 (0.63–1.34)	< 0.001⁣^∗∗^
Blood sedimentation (mm/h)	56.0 (34.0–75.0)	56.5 (34.5–73.5)	55.0 (30.0–80.0)	0.84
C-reactive protein (mg/L)	45.2 (17.4–101.0)	90.2 (34.5–134.0)	19.9 (11.6–45.2)	< 0.001⁣^∗∗^
Procalcitonin (ng/mL)	1.49 (0.44–5.73)	3.14 (0.56–14.19)	0.65 (0.37–1.49)	< 0.001⁣^∗∗^

Abbreviations: AKI, acute kidney injury; CKD, chronic kidney disease; CRRT, continuous renal replacement therapy; IHD, intermittent hemodialysis.

⁣^∗^*p* < 0.05.

⁣^∗∗^*p* < 0.01.

**Table 2:** Treatment of patients in the CRRT and IHD groups.

Variant	Total (*n* = 216)	CRRT group (*n* = 119)	IHD group (*n* = 97)	*p* value
Vascular access (*n*, %)				
Jugular vein cannulation	108 (50.0)	15 (12.6)	93 (95.9)	< 0.001⁣^∗∗^
Femoral vein cannulation	108 (50.0)	104 (87.4)	4 (4.1)	
Anticoagulation modalities (*n*, %)				
Heparin-free	52 (24.1)	22 (18.5)	30 (31.3)	
Low-molecular heparin	89 (41.2)	31 (26.1)	58 (59.4)	< 0.001⁣^∗∗^
Sodium citrate	75 (34.7)	66 (55.5)	9 (9.4)	
Filter Coagulation (*n*, %)	33 (15.2)	21 (17.6)	12 (12.1)	0.449
Filter allergy (*n*, %)	13 (6.0)	8 (6.7)	5 (5.2)	0.625
RRT mode switching (*n*, %)	24 (11.1)	19 (16.0)	5 (5.2)	0.012⁣^∗^
RRT duration (h)	47 (36–55)	52 (34–81)	17 (11–24)	0.002⁣^∗∗^
RRT cost (yuan)	7091 (5411–8530)	8382 (5712–13770)	2205 (1200–3100)	< 0.001⁣^∗∗^
Length of hospitalization (h)	384 (264–618)	456 (261–760)	360 (264–552)	0.012⁣^∗^
Survival status at discharge (*n*, %)				
Death	61 (28.2)	59 (49.6)	2 (2.1)	< 0.001⁣^∗∗^
Survival	155 (71.8)	60 (50.4)	95 (97.9)	

Abbreviations: CRRT, continuous renal replacement therapy; IHD, intermittent hemodialysis.

⁣^∗^*p* < 0.05.

⁣^∗∗^*p* < 0.01.

**Table 3:** Baseline characteristics of patients with and without recovered renal function at discharge.

Variant	Total (*n* = 155)	Recovery of renal function (*n* = 77)	No recovery of renal function (*n* = 78)	*p* value
Male (*n*, %)	104 (67.1)	49 (63.6)	55 (70.5)	0.362
Age (years)	66 (63–72)	65 (62–71)	69 (64–72)	0.001⁣^∗∗^
Section source (*n*, %)				
Internal medicine	87 (56.1)	34 (44.2)	53 (67.9)	0.009⁣^∗∗^
Surgery	8 (5.2)	7 (9.1)	1 (1.3)	
Emergency	26 (16.8)	17 (22.1)	9 (11.5)	
ICU	34 (21.9)	19 (24.7)	15 (19.2)	
Underlying disease (*n*, %)				
Hypertension	97 (62.6)	46 (59.7)	51 (65.4)	0.468
Diabetes mellitus	30 (19.4)	14 (18.2)	16 (20.5)	0.713
Cardiovascular disease	64 (41.3)	26 (33.8)	38 (48.7)	0.059
CKD	53 (34.2)	18 (23.4)	35 (44.9)	0.005⁣^∗∗^
Smoking history (*n*, %)	66 (42.6)	30 (39.0)	36 (46.2)	0.365
Drinking history (*n*, %)	37 (23.9)	16 (20.8)	2 (26.9)	0.370
Baseline blood creatinine (μmol/L)	93 (84–160)	92 (75–142)	103 (88–203)	0.011⁣^∗^
AKI pathogenic factors (*n*, %)				
Insufficient renal perfusion	23 (14.8)	14 (18.2)	9 (11.5)	0.095
Primary or secondary glomerular disease	49 (31.6)	19 (24.7)	30 (38.5)	
Post-surgical kidney injury	10 (6.5)	4 (5.2)	6 (7.7)	
Drug-induced renal injury	31 (20.0)	21 (27.3)	10 (12.8)	
Severe infection or sepsis	25 (16.1)	13 (16.9)	12 (15.4)	
Other	17 (11.0)	6 (7.8)	11 (14.1)	
AKI stages (*n*, %)				
Stage 1	25 (16.1)	11 (14.3)	14 (17.9)	0.787
Stage 2	25 (16.1)	12 (15.6)	13 (16.7)	
Stage 3	105 (67.7)	54 (70.1)	51 (65.4)	
APACHE II score (points)	15 (13–24)	14 (11–23)	18 (16–27)	0.540
Cardiovascular accident (*n*, %)	23 (14.8)	9 (11.7)	14 (17.9)	0.273
Vasoactive drug (*n*, %)	17 (11.0)	7 (9.1)	10 (12.8)	0.458
Mechanical ventilation (*n*, %)	27 (17.4)	14 (18.2)	13 (16.7)	0.804
Urination (*n*, %)				
Anuria	48 (31.0)	24 (31.2)	24 (30.8)	0.754
Oliguria	71 (45.8)	37 (48.1)	34 (43.6)	
Normal	36 (23.2)	16 (20.8)	20 (25.6)	
Leukocyte (× 10^9^/L)	9.3 (6.9–13.0)	9.5 (7.5–13.0)	8.6 (6.7–13.0)	0.239
Hemoglobin (g/L)	98.5 ± 25.3	106.6 ± 24.4	90.6 ± 23.7	< 0.001⁣^∗∗^
Blood platelet (× 10^9^/L)	173 (107–237)	170 (105–216)	176 (107–242)	0.625
Neutrophil (× 10^9^/L)	7.1 (5.3–11.0)	8.0 (5.5–11.0)	6.5 (5.2–10.9)	0.362
Lymphocyte (× 10^9^/L)	0.9 (0.6–1.2)	0.9 (0.6–1.2)	0.8 (0.5–1.2)	0.415
Hematuria (*n*, %)	92 (59.4)	45 (58.4)	47 (60.3)	0.818
Proteinuria (*n*, %)	111 (71.6)	52 (67.5)	59 (75.6)	0.263
Albumin (g/L)	30.1 ± 6.7	31.0 ± 6.7	29.2 ± 6.6	0.097
Glutamine aminotransferase (U/L)	19.5 (10.1–47.0)	21.7 (10.1–59.0)	16.9 (10.2–37.2)	0.284
Glutamic transaminase (U/L)	31.3 (19.1–59.0)	32.4 (19.9–70.7)	28.3 (18.8–45.1)	0.209
Urea nitrogen (mmol/L)	23.7 (16.1–36.2)	22.1 (16.6–32.9)	25.6 (15.9–36.6)	0.639
Serum creatinine (μmol/L)	622 ± 330	617 ± 335	626 ± 326	0.895
Uric acid (μmol/L)	549 (391–676)	549 (382–703)	547 (433–634)	0.947
Serum potassium (mmol/L)	4.36 (3.88–4.92)	4.26 (3.84–4.88)	4.42 (3.91–4.97)	0.450
Blood sodium (mmol/L)	139 (136–143)	139 (135–142)	140 (137–143)	0.250
Blood chloride (mmol/L)	102.3 (96.8–105.8)	102.1 (96.6–105.0)	102.9 (97.6–106.8)	0.354
Anion gap (mmol/L)	18.15 ± 5.42	18.72 ± 5.60	17.59 ± 5.21	0.196
Blood glucose (mmol/L)	5.77 (4.75–7.61)	5.85 (4.75–7.52)	5.54 (4.76–7.61)	0.916
Triglyceride (mmol/L)	1.50 (1.14–2.18)	1.50 (1.15–2.08)	1.50 (1.12–2.19)	0.575
Cholesterol(mmol/L)	3.94 (3.10–5.11)	3.80 (3.21–5.38)	3.95 (3.10–4.69)	0.637
High density lipoprotein (mmol/L)	0.93 (0.75–1.22)	0.97 (0.77–1.23)	0.91 (0.75–1.20)	0.425
Low density lipoprotein (mmol/L)	2.47 (2.00–3.23)	2.35 (2.02–3.33)	2.55 (1.97–3.16)	0.706
Lactate dehydrogenase (U/L)	345.2 (234.0–420.9)	345.9 (260.0–441.0)	336.5 (199.0–417.0)	0.315
Creatine kinase (U/L)	103.0 (55.0–172.0)	93.9 (50.0–195.3)	103.0 (61.1–164.5)	0.533
Creatine kinase isoenzyme (U/L)	17.1 (10.9–23.5)	17.1 (11.8–24.3)	16.5 (10.9–21.0)	0.498
Myoglobin (μg/L)	213.9 (80.9–276.6)	166.3 (74.8–238.5)	219.1 (101.6–295.5)	0.078
Prothrombin time (s)	13.5 (12.3–14.7)	13.6 (12.7–15.2)	13.4 (12.2–14.7)	0.210
Activated partial thromboplastin time (s)	35.6 (30.3–42.8)	35.8 (30.3–43.3)	35.3 (30.3–41.6)	0.745
D-dimer (mg/L)	1.34 (0.63–2.07)	1.34 (0.63–1.54)	1.34 (0.74–2.45)	0.225
Blood sedimentation (mm/h)	56.0 (34.0–75.0)	56.0 (38.0–71.0)	56.0 (30.0–78.0)	0.580
C-reactive protein(mg/L)	43.7 (15.1–67.4)	45.20 (19.20–63.10)	25.40 (8.94–67.40)	0.157
Procalcitonin (ng/mL)	1.49 (0.37–3.24)	1.49 (0.68–3.80)	1.49 (0.30–3.14)	0.242

⁣^∗^*p* < 0.05.

⁣^∗∗^*p* < 0.01.

**Table 4:** Treatment of patients with and without recovery of renal function at hospital discharge.

Variant	Total (*n* = 155)	Recovery of renal function (*n* = 77)	No recovery of renal function (*n* = 78)	*p* value
RRT mode (*n*, %)				
IHD	95 (61.3)	46 (59.7)	49 (62.8)	0.694
CRRT	60 (38.7)	31 (40.3)	29 (37.2)	
Vascular access (*n*, %)				
Jugular vein cannulation	102 (65.8)	51 (66.2)	51 (65.4)	0.911
Femoral vein cannulation	53 (34.2)	26 (33.8)	27 (34.6)	
Anticoagulation modalities (*n*, %)				
Heparin-free	18 (11.6)	8 (10.4)	10 (12.8)	0.842
Low-molecular heparin	103 (66.5)	51 (66.2)	52 (66.7)	
Sodium citrate	34 (21.9)	18 (23.4)	16 (20.5)	
Filter Coagulation (*n*, %)	13 (8.4)	5 (6.5)	8 (10.3)	0.398
Filter allergy (*n*, %)	6 (3.9)	1 (1.3)	5 (6.4)	0.099
RRT mode switching (*n*, %)	21 (13.5)	9 (11.7)	12 (15.4)	0.501
RRT duration (h)	39 (37–44)	37 (30–43)	43 (41–47)	0.043⁣^∗^
RRT cost (yuan)	5883 (5114–6893)	5582 (4740–6487)	6487 (5781–7386)	0.053
Length of hospitalization (h)	384 (264–600)	360 (288–600)	431 (264–600)	0.900

⁣^∗^*p* < 0.05.

**Table 5:** Univariate analysis of covariates that have an impact on the recovery of renal function at discharge in elderly AKI patients.

Variant	*p value*	OR value	95% CI
Age	0.009⁣^∗∗^	1.073	1.018–1.130
CKD	0.005⁣^∗∗^	2.668	1.337–5.325
Baseline creatinine	0.026⁣^∗^	1.004	1.000–1.007
Hemoglobin (per 10 g/L increase)	< 0.001⁣^∗∗^	0.760	0.660–0.875

Abbreviations: AKI, acute kidney injury; CI, confidence interval; CKD, chronic kidney disease; OR, odds ratio.

⁣^∗^*p* < 0.05.

⁣^∗∗^*p* < 0.01.

**Table 6:** Multifactorial logistic regression analysis of factors influencing the recovery of renal function in elderly patients with AKI at discharge.

Variant	Model 1			Model 2		
	*p* value	OR value	95% CI	*p* value	OR value	95% CI
RRT mode						
IHD (reference)						
CRRT	0.125	0.528	0.234–1.193	0.124	0.521	0.226–1.197
Age	0.021⁣^∗^	1.069	1.010–1.130			
CKD	0.017⁣^∗^	2.573	1.186–5.585			
Baseline creatinine				0.118	0.997	0.993–1.001
AKI stages						
Stage 1 (reference)						
Stage 2	0.994	0.996	0.289–3.435	0.884	1.099	0.308–3.925
Stage 3	0.940	0.958	0.316–2.908	0.837	0.888	0.286–2.758
Hemoglobin (per 10 g/L increase)				< 0.001⁣^∗∗^	0.750	0.644–0.873

Abbreviations: AKI, acute kidney injury; CI, confidence interval; CKD, chronic kidney disease; CRRT, continuous renal replacement therapy; IHD: intermittent hemodialysis; OR, odds ratio; RRT, renal replacement therapy.

⁣^∗^*p* < 0.05.

⁣^∗∗^*p* < 0.01.

**Table 7:** Univariate analysis of covariates that have an impact on maintenance dialysis in elderly AKI patients.

Variant	*p* value	OR value	95% CI
RRT mode			
IHD (reference)			
CRRT	0.045⁣^∗^	0.286	0.084–0.971
Age	0.021⁣^∗^	1.111	1.016–1.215
White blood cell count	0.025⁣^∗^	0.863	0.759–0.982

Abbreviations: AKI: acute kidney injury; CI: confidence interval; CRRT: continuous renal replacement therapy; IHD: intermittent hemodialysis; OR: odds ratio; RRT: renal replacement therapy.

⁣^∗^*p* < 0.05.

**Table 8:** Multifactorial logistic regression analysis of factors affecting maintenance dialysis in elderly AKI patients.

Variant	Model 1			Model 2		
	*p* value	OR value	95% CI	*p* value	OR value	95% CI
RRT mode						
IHD (reference)						
CRRT	0.033⁣^∗^	0.124	0.018–0.847	0.010⁣^∗^	0.098	0.017–0.571
AKI stages						
Stage 1 (reference)						
Stage 2	0.628	1.972	0.126–30.776			
Stage 3	0.625	1.681	0.209–13.51			
Age	0.018⁣^∗^	1.149	1.024–1.289	0.013⁣^∗^	1.146	1.029–1.277
CKD	0.051	4.249	0.992–18.2	0.058	4.286	0.953–19.282
White blood cell count				0.074	0.867	0.741–1.014

Abbreviations: AKI: acute kidney injury; CI: confidence interval; CKD: chronic kidney disease; CRRT: continuous renal replacement therapy; IHD: intermittent hemodialysis; OR: odds ratio; RRT: renal replacement therapy.

⁣^∗^*p* < 0.05.

**Table 9:** Univariate analysis of covariates influencing renal function recovery at discharge in non-CKD elderly patients with AKI.

Variant	*p* value	OR value	95% CI
Age	0.007⁣^∗^	1.107	1.028–1.192
Hemoglobin (per 10 g/L increase)	0.005⁣^∗^	0.789	0.668–0.931

Abbreviations: AKI: acute kidney injury; CI: confidence interval; CKD: chronic kidney disease; OR: odds ratio.

⁣^∗^*p* < 0.01.

**Table 10:** Multifactorial logistic regression analysis of factors influencing renal function recovery at discharge in non-CKD-elderly AKI patients.

Variant	*p* value	OR value	95% CI
Age	0.021⁣^∗^	1.098	1.014–1.190
RRT mode			
IHD (reference)			
CRRT	0.143	0.459	0.162–1.303
AKI stages			
Stage 1 (reference)			
Stage 2	0.176	3.929	0.541–28.528
Stage 3	0.748	1.323	0.240–7.278
Hemoglobin (per 10 g/L increase)	0.015⁣^∗^	0.796	0.622–0.958

Abbreviations: AKI: acute kidney injury; CI: confidence interval; CKD: chronic kidney disease; CRRT: continuous renal replacement therapy; IHD: intermittent hemodialysis; OR: odds ratio; RRT: renal replacement therapy.

⁣^∗^*p* < 0.05.

## Data Availability

The data that support the findings of this study are available from the corresponding author upon reasonable request.
